# Antioxidant, Antimicrobial Activities and Fatty Acid Compositions of Wild *Berberis* spp. by Different Techniques Combined with Chemometrics (PCA and HCA)

**DOI:** 10.3390/molecules26247448

**Published:** 2021-12-09

**Authors:** Betül Gıdık

**Affiliations:** Department of Organic Farming Management, Bayburt University, Bayburt 69000, Turkey; betulgidik@bayburt.edu.tr

**Keywords:** bioactivity, medicinal plants, wild fruits, industrial crops, PCA, HCA

## Abstract

Interest in medicinal plants and fruits has increased in recent years due to people beginning to consume natural foods. This study aims to investigate the total phenolic flavonoid content, antioxidant activity, condensed tannin content, oil content, and fatty acid compositions of five local breeds of *Berberis* spp. from Bayburt, Turkey, and their antioxidant and antimicrobial activities. The fatty acid composition of samples was performed with gas chromatography-mass spectrometry (GC-MS), and the total fatty acid content of samples was between 6.12% and 8.60%. The main fatty acids in *Berberis* spp. samples were α-linolenic acid (32.85–37.88%) and linoleic acid (30.98–34.28%) followed by oleic acid (12.85–19.56%). Two antioxidant assays produced similar results, demonstrating that extracts of wild *B. vulgaris* L. had the highest ferric reducing antioxidant power (FRAP) (621.02 μmol FeSO_4_.7H_2_O/g) and 1,1-diphenyl-2-picrylhydrazyl radical (DPPH) (0.10 SC_50_ mg/mL) values. According to principal component analysis (PCA), four components were determined. In addition, two main groups were determined according to hierarchical cluster analysis (HCA), and wild and culture of *B. vulgaris* L. were in different subgroups. This is the first original report about the fatty acid composition and oil content of *Berberis* spp. grown in Bayburt, Turkey. The obtained results indicate that *B. integerrima* Bunge and *B. vulgaris*, which have especially remarkable fatty acid content, antioxidant, and antimicrobial activity, could be potential sources for these properties in different areas of use.

## 1. Introduction

The increasing world population has caused people to search for new food sources. Therefore, wild plants are gaining increasing value. Many scientific studies have been published on the nutritional content and medicinal values of wild edible fruits grown in various parts of the World [1,2]. Interest in wild plants has increased in recent years since people have recently begun consuming natural foods. For this reason, the nutrient content of wild plants should be determined and those that can be considered as a food source should be determined and their cultivation should be encouraged together with breeding studies. In fact, local people in different countries still use the fruits of wild plants to protect their health, and the pharmacological properties of these wild fruits are attributed to phenolic compounds that act as natural antioxidants [3]. When evaluated in this respect, phenolic compounds are antioxidant molecules found in all plants and whose bioactive properties are well known. Therefore, in recent years, research has focused on the identification and measurement of phenolic compounds as medicinal and food molecules in natural plant sources, especially wild plants. The protection provided by the consumption of plant products such as fruits, vegetables, and legumes is mostly related to the presence of phenolic compounds [4]. In addition, due to the increasing use of these compounds in industrial areas (development of functional food and nutraceuticals, etc.), it is very important to determine the natural sources.

The Berberidaceae family contains about 14 genera and 700 species all over the world. In addition, four wild species grow in Turkey: *B. vulgaris* L., *B. cretica* L., *B. crataegina* DC., and *B. integerrima* Bunge [5]. *Berberis* spp. fruits can be eaten raw or cooked and have been used for ornamental purposes, medical as well as food additives, especially in the form of dried fruit [6]. In addition, its rich vitamin content and highly acidic fruits are used to increase the body’s resistance. Dried fruits are used as an additive in food and meals, while fresh fruits are used for making jelly, jam, syrup, sauce, fruit juice, and carbonated beverages. However, in recent years, fruits have been used as a colorant in the food industry due to their anthocyanin content [6,7,8].

*Berberis* spp. is becoming increasingly important because due to its bioactive properties such as antioxidants, antimicrobials, etc. [9]. For medical purposes, some studies have been carried out on the fruit and root of *Berberis vulgaris*. Berberine, Vitamin C, different vitamins, and salt are known to be isolated from the fruit, root, and peel of *B. vulgaris* [10]. Different parts of *Berberis* spp. plants have a wide range of phenolic compounds, vitamins, and some other metabolites, which are rich in antioxidant activity [11]. Some studies have been put forth that *Berberis* spp. contain the phenols DPPH and FRAP [12,13].

Oils are one of the essential nutrients for humans, and fatty acids form the basis of them. Fatty acids are grouped according to the availability of saturated, monounsaturated, and polyunsaturated fatty acids [14,15]. In addition, an average of 200 fatty acids are detected, and vegetable oils are known to be rich in oleic and linoleic fatty acids [16]. There are few studies published on the fatty acid composition of seeds of *Berberis* spp. [17,18].

In this study, for the first time, the oil content and fatty acid compositions of seeds of wild *Berberis vulgaris* L., *Berberis integerrima* Bunge, *Berberis crataegina* DC., the culture form of *Berberis vulgaris* L., and the hybrid of *Berberis integerrima* × *Berberis crataegina* was determined. With this, total flavonoid content, total phenolic content, and antioxidant activities were determined in order to gain an indication about the bioactive content of the samples. In addition, in vitro antibacterial activities of the samples were determined by agar well diffusion, minimum inhibition concentration (MIC), and minimum bactericidal concentration (MBC) techniques. 

## 2. Results and Discussion

Although plants have been at the forefront of medicinal uses for thousands of years, the active ingredients of many plants are used in the production of drugs in modern technology. For this reason, it is important to determine the potential bioactive properties of different plant sources and, in addition, the bioactive active substances in detail. Especially in recent years, depending on the increase in the world population, the chemical contents of wild plants consumed by local people in different nations are a matter of curiosity, but the amount of research on this subject is limited. Therefore, in this study, some bioactive properties of different *Berberis* species—about which there was limited research before—were elucidated. For *Berberis* spp. used in the study, the total oil content of the samples and also 20 different fatty acids were screened. As a result, the oil content of *Berberis* spp. changed between 6.12% and 8.60% (Table 1). According to this, *B. crataegina* DC. had the lowest ratio and the wild *B. vulgaris* had the highest one. Similarly, the amount of *Berberis integerrima* seed oil content was found to be lower than the results reported in the literature [19]. This situation is thought to be due to the ecological differences between the collecting locations. However, when the individual fatty acid composition of *Berberis* spp. was examined, except for four fatty acids (eicosadienoic acid, arachidonic acid, tricosanoic acid, and nervonic acid), the others were detected at different rates. It is seen that the major fatty acids were α-linolenic acid (32.85 ± 3.31–37.88 ± 1.71), linoleic acid (30.98 ± 1.46–34.28 ± 1.84), and oleic acid (12.85 ± 2.88–19.56 ± 3.88). Fatty acids contain one or more covalent double bonds between carbon-carbon at various positions on the carbon chain are called unsaturated fatty acids. While oleic acid is in the group of monounsaturated fatty acids, linoleic and linolenic acids are among the polyunsaturated fatty acids. The highest amounts of α-linolenic acid, linoleic acid, and oleic acid methyl ester were determined in *B. crataegina* DC., *B. integerrima* Bunge, and a hibrid of *B. integerrima* × *B. crataegina*, respectively. Our results were found to be higher than the studies by other researchers [20]. The difference may be related to the diversity of *Berberis* species, as well as many factors that affect the development of the plant, such as the geographical characteristics of the cultivation area where the plants grow, soil characteristics. 

Fatty acids that consist of a single covalent bond between carbon-carbon atoms and are generally solid at room temperature are called saturated fatty acids. Lauric acid (C12:0), palmitic acid (C16:0), myristic acid (C14:0), stearic acid (C18:0), behenic acid (C22:0), and arachidic acid (C20:0) found in vegetable oils are the most important saturated fatty acids. Fatty acids that contain one or more covalent double bonds between carbon-carbon atoms at various positions on the carbon chain are called unsaturated fatty acids [21]. Saturated fatty acids can be synthesized in the human body, and even if no fat is consumed, these types of fatty acids can be synthesized from molecules formed by carbohydrate metabolism. In response to this, unsaturated fats are essential fatty acids that the body needs. They are liquid at room temperature and most of them are of vegetable origin [21,22]. In addition, saturated fatty acids were found at 14.38% and 16.42% total amount, while unsaturated fatty acids were found between 84.16% and 85.62. The values were changed for the saturated/unsaturated fatty acids ratio at 0.17% to 0.20%, for monounsaturated fatty acids at 13.03% to 19.64, and for polyunsaturated fatty acids at 63.94% to 71.43% (Table 2). The results for the saturated/unsaturated fatty acids ratio were similar to some studies [16,23] in the literature. The saturated fatty acid results in this study were higher than those reported by Kaya et al. [20], but they also had monounsaturated fatty acids similar to them. Similarities showed that, in general, the *Berberis* spp. seeds had the same saturated and unsaturated fatty acids ratios.

Antimicrobials play an important role in transporting foodstuffs over long distances or extending shelf life. It is thought that antimicrobial compounds obtained from plants can be a healthy alternative in food preservation [24]. The plants that belong to the Berberidaceae were effective in many analyses employed for antioxidant and antimicrobial activity [25,26,27]. Our results showed that *Berberis* spp. fruit extracts have, in general, an antibacterial effect against all Gram-positive and Gram-negative bacteria at a concentration of 128 mg/mL. When we evaluated species in terms of antibacterial activity, it was observed that *B. integerrima* × *B. crataegina*, *B. integerrima* Bunge, *B. vulgaris* L. (culture), and *B. vulgaris* L. (wild) species had an in vitro inhibitory effect against all selected pathogens. However, it was observed that *B. crataegina* DC. had antibacterial effects against nine bacterial strains from target pathogens, while it did not have antibacterial effects against the remaining nine samples. In addition, when the inhibition zone diameter was evaluated, it was observed that *B. crataegina* DC. had inhibition zone diameters ranging from 10 to 17 mm, and it was observed to have a weaker inhibition effect than other samples. In addition, it has been observed that wild *B. vulgaris* L. generally has a larger inhibition zone diameter compared to other samples, and zone diameters range from 18 to 39 mm. The results of Aliakbarlu et al. [28] were similar to ours, however, Irshad et al. [29] found lower values for inhibition zone diameters of *Berberis* spp. The differences may be caused by the use of different extraction methods or solvents. On the other hand, the species can be effective at these differences. As in previous similar studies, in the results obtained in this study, *Berberis* spp. was found to be suitable for use as a food. In addition, it is thought that it will be an important source for more comprehensive studies to be conducted for *Berberis* spp., whose antimicrobial properties are determined.

Among the Gram-positive bacteria, *Bacillus cereus* ATCC 14579, *Bacillus cereus* BC 6830, *Enterococcus faecalis* ATCC 49452, *Enterococcus faecalis* NCTC 12697, *Streptococcus mutans* ATCC 35668, and *Streptococcus salivarus* ATCC 13419 strains were found to be the most susceptible strains against *Berberis* spp. fruit extracts. It was observed that these strains were sensitive to all extracts, including *B. crataegina* DC. In addition, it was observed that *Enterococcus faecium* ATCC 700211, *Staphylococus aureus* ATCC 25923, *Staphylococcus aureus* NCTC 10788, and *Staphylococcus aureus* BC 7231 strains were resistant to *B. crataegina* DC. (Table 3). The obtained results showed that *Berberis* spp. fruit samples have an antibacterial effect. In addition, it is concluded that it may be beneficial to use these fruits as food supplements for phytotherapy. 

Antioxidants are bodily defense mechanisms developed to prevent damage caused by the formation of reactive oxygen species (ROS). Antioxidants are substances that prevent the deterioration of the structural and functional molecules in the body, especially lipid, protein, carbohydrates, and DNA, and are effective against free radicals even at low concentrations [30]. Studies in this area of *Berberis* spp. showed that plants are valuable in terms of antioxidant content and these plants are edible. Results of the total TP, TF, CT, FRAP, and DPPH assay of *Berberis* fruits are presented in Table 4. The total phenolic content of the samples was observed to be within the range of 10.84–28.92 mg GAE/g, with the lowest and highest levels observed in *B. crataegina* DC. and *B. vulgaris* L. (wild) samples, respectively. Previous studies also revealed the total phenolic content of *Berberis* spp. fruits, and our results were lower [13,31,32] or higher [24]. TF and CT were found between the range of 0.41–2.20 mg QE/g and 1.75–6.92 CE/g, respectively. *B. crataegina* DC. has the highest value and the results were similar to those of Dimitrijević et al. [13]. FRAP and DPPH were detected in the order of 218.55–621.02 μmol FeSO_4_.7H_2_O/g and 0.10–0.36 mg/mL; *Berberis vulgaris* L. (wild) has the highest level of FRAP and the lowest level of DPPH. Previous studies found lower FRAP and DPPH than our results [13,24] or similar results from Koncˇic’ et al. [27]. There is a difference, which, similar to other studies, shows that it can be influenced by geographical conditions and the significance of the samples’ origin. Total TP, TF, CT, FRAP, and DPPH values were found to be among the values considered suitable for food use for *Berberis* spp. and especially important among wild plants.

A Pearson correlation analysis was performed to determine the connection between the variables. To determine the relationship between the antioxidant activities of *Berberis* spp. and their oil content, a Pearson correlation analysis was performed. According to the Pearson correlation, a positive correlation was found between TP and FRAP (*p* = 0.02), and CT with DPPH (*p* = 0.02) at *p* < 0.05 level. In addition, a positive correlation was determined between CT and TF (*p* = 0.00) at a *p* < 0.01 level.

While a negative correlation was found between oil content and TF (*p* = 0.04) at a *p* < 0.05 level, negative correlations were found in oil content either with CT (*p* = 0.01) or DPPH (*p* = 0.00) at a *p* < 0.01 level. The correlation results showed that oil content was negatively affected by the amount of TF, CT, and DPPH. On the other hand, TP and FRAP, TF and CT, DPPH and CT, were affected positively. As revealed by the Pearson correlation analysis, although there is a negative interaction between fat ratio and TF, CT, DPPH, the obtained fat ratio and TF, CT, DPPH values show that *Berberis* spp. is suitable for consumption as food in daily life.

**Figure 1:**
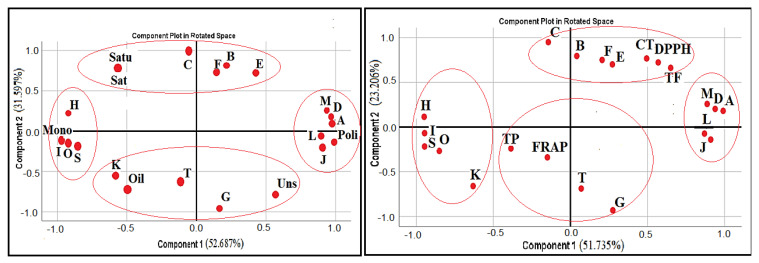
PCA-1 and PCA-2 score plot bases on the fatty acid composition with oil contents and the fatty acids together with antioxidant parameters. Abbreviations and labels: (**A**) Butyric acid methyl ester, (**B**) caproic acid methyl ester, (**C**) undecanoic acid methyl ester, (**D**) lauric acid methyl ester, (**E**) myristic acid methyl ester, (**F**) palmitic acid methyl ester, (**G**) heptadecanoic acid methyl Eeter, (**H**) stearic acid methyl ester, (**I**) oleic acid methyl ester, (**J**) linoleic acid methyl ester, (**K**) arachidic acid methyl ester, (**L**) α-linolenic acid methyl ester, (**M**) cis-11-eicosenoic acid methyl ester, (**O**) behenic acid methyl ester, (**S**) *cis*-58111417 eicosapentaenoic acid methyl ester, (**T**) lignoceric acid methyl ester, (**Sat**) saturated fatty acids, (**Uns**) unsaturated fatty acids, (**Satu**) saturated/unsaturated fatty acid ratio, (**Mono**) monounsaturated fatty acids, (**Poli**) polyunsaturated fatty acids, (**Oil**) oil content, (**TP**) total phenolic content, (**TF**) total flavonoid content, (**CT**) condensed tannin content, (**FRAP**) ferric reducing antioxidant power, (**DPPH**) 1,1-Diphenyl-2-picrylhydrazyl radical.

PCA is known as a method that reveals the variance structure of the original *p* variable with fewer new variables that are the linear components of the variables. According to PCA1, four components were determined for fatty acids and saturation of oil content. Eigenvalues and variance percentages of PCA1 and PCA2 analysis are provided in Table 5 and graphs are provided in Figure 1. It is indicated that variance explanation ratios over 70% were sufficient in the PCA analysis [33]. For PCA1, PC1 and PC2 explained 84.28% of the total variation, while PC1 explained 52.69% and PC2 explained 31.60% of the total variation. In PCA2, PC1 explained 51.73%, and PC2 explained 23.21%, and the total variation explained 74.94%. According to Kaiser rules, eigenvalues of greater than 1.0 are accepted as the principal component and descriptor of the variance [34]. In PCA1, eigenvalues of PC1 (11.59), PC2 (6.95), PC3 (2.29) and PC4 (1.16) were greater than 1.0. In PCA2, eigenvalues of PC1 (10.86), PC2 (4.87), PC3 (3.68) and PC4 (1.58) were greater than 1.0.

The Kaiser–Meyer–Olkin (KMO) test was used to measure sampling adequacy in principal component analysis. According to Kaiser [34], the KMO coefficient is unacceptable between 0–0.5, 0.5 is the minimum, between 0.5–0.7 is the medium, between 0.7–0.8 is good, 0.8–0.9 is considered very good, and 0.9 and above is considered perfect. According to this, PC1 was found to be related to butyric acid methyl ester, lauric acid methyl ester, stearic acid methyl ester, oleic acid methyl ester, linoleic acid methyl ester, arachidic acid methyl ester, α-linolenic acid methyl ester, cis11-eicosenoic acid methyl ester, behenic acid methyl ester, *cis*-58111417-eicosapentaenoic acid methyl ester, saturated fatty acids, unsaturated fatty acids, saturated/unsaturated fatty acid ratio, monounsaturated fatty acids, polyunsaturated fatty acids, and oil content, were indexed in PCA1 and found to be related to butyric acid methyl ester, cis58111417 eicosapentaenoic acid methyl ester, lauric acid methyl ester, cis11 eicosenoic acid methyl ester, TF, arachidic acid methyl ester, oleic acid methyl ester, behenic acid methyl ester, DPPH, CT, stearic acid methyl ester, α-linolenic acid methyl ester, linoleic acid methyl ester, palmitic acid methyl ester, and myristic acid methyl ester, which were indexed in PCA2. 

**Figure 2:**
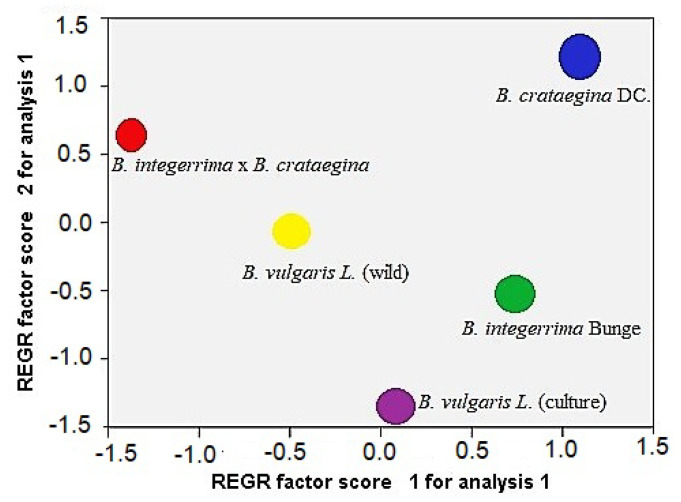
Distribution of the species according to the main components.

PCA1 and PCA2 graphs for fatty acid composition and fatty acid composition with antioxidant parameters are presented in Figure 1. Moreover, the score plots of the species distribution according to the main components are shown in Figure 2. The score plot shows the differences between species. Although wild and culture forms of *B. vulgaris* are the same species, there are some differences between them. Moreover, the hybrid of *B. integerrima* × *B. crataegina* is not the same as either *B. integerrima* or *B. crataegina*. All these results are supported by our determinations. According to the results obtained, the presence of both the fatty acids and TF, TP, CT, DPPH, and FRAP values among all the basic components, and that these values determined in *Berberis* spp., underline the need for further study.

**Figure 3:**
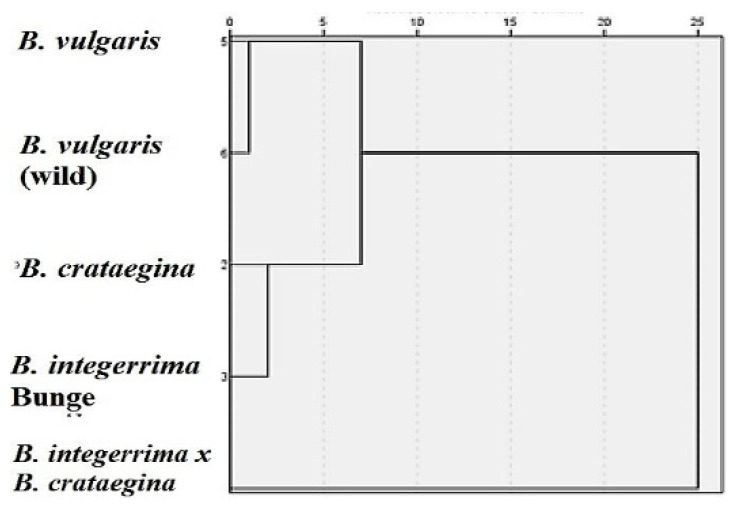
The HCA dendrogram according to the fatty acid compositions of *Berberis* spp.

HCA is a clustering method that explores the organization of samples. Furthermore, it allows for the determination of similarities and differences within and between groups by depicting a hierarchy [35]. The results of HCA are generally presented in a dendrogram—a plot that shows the organization of samples and their relationships in tree form. There are two common approaches to resolve the grouping problem in HCA: divisive and agglomerative. According to the HCA of *Berberis* spp., two main groups were determined (Figure 3).

The first one occurred in *B. integerrima* × *B. crataegina*. The second main group contained two subgroups that were separated from each other. Although culture *B. vulgaris* and wild *B. vulgaris* were in a close subgroup, they were separated from each other. These results indicate that culture and wild form plants cannot be the same. Although *Berberis* spp. are closely related plants, it has been observed that they differ in oil ratios, fatty acid compositions, antioxidant, and antimicrobial properties.

The usage areas of medicinal aromatic and wild plants are expanding day by day. There are issues in this field that have not yet been sufficiently clarified. *Berberis* spp. is among plants that grow wild in the Bayburt region of Turkey and are consumed as food by the public. No study has covered all wild *Berberis* spp. naturally grown in this region. This study, which included all wild *Berberis* spp. in the Bayburt region of Turkey, showed that it is suitable for consumption as food and can be used with different food products.

## 3. Materials and Methods

### 3.1. Plant Material

Five different local breeds of *Berberis* sp. (wild *Berberis vulgaris* L., *Berberis integerrima* Bunge, *Berberis crataegina* DC., the culture form of *Berberis vulgaris* L., and the hybrid of *Berberis integerrima* × *Berberis crataegina*) were collected from Bayburt, an eastern Black Sea city in Turkey, during September 2019. The locations were: Kop Mountain Pass, Sancaktepe and Demirozu crossroads, Sirakayalar Village, and Eski Kopuz Village Road, respectively. The altitude, latitude, and longitude data that belong to the collection location of wild *B. vulgaris* L. 2204 m, 40°03′11″ N, 40°28′42″ E, the culture form of *B. vulgaris* L. 1675 m, 40°13′22″ N, 40°03′51″ E, *B. integerrima* Bunge 1882 m, 40°06′01″ N, 40°14′03″ E, *B. crataegina* DC. 1579 m, 40°12′41″ N, 40°15′42″ E, and the hybrid of *B. integerrima* × *B. crataegina* 1592 m, 40°12′01″ N, 40°17′22″ E. 

**Figure 4:**
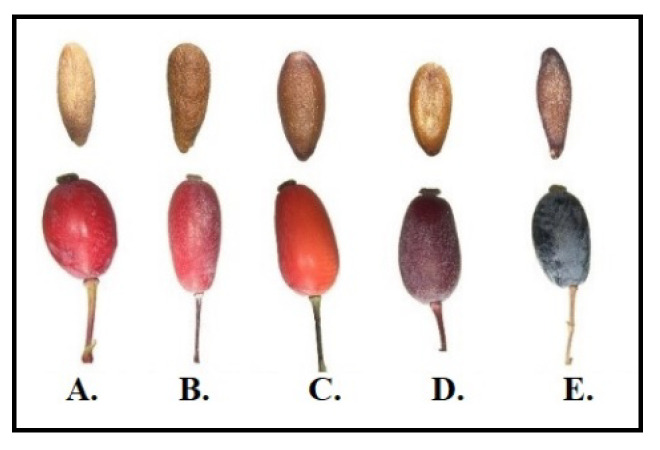
The fruits and seeds of *Berberis* spp. (**A**) *B. vulgaris* L., (**B**) *B. vulgaris* L. *, (**C**) *B. integerrima* Bunge, (**D**) *B. integerrima* × *B. crataegina* **, (**E**) *B. crataegina* DC., * Culture plant ** Hybrid.

Leaves and stems were separated from fruits, and after drying for two months at room temperature and were stored in a dry environment until analyzed. The fruits and seeds of the *Berberis* sp. were photographed by a binocular microscope to support the taxonomic classification. The fruits and seeds belonging to different *Berberis* spp. are shown in Figure 4.

### 3.2. Extraction of Plant Material

Ultrasonic assisted extraction of *Berberis* spp. fruit samples was performed with minor modifications from methods described by Annegowda et al. [36] and Wang et al. [37]. *Berberis* fruit samples were stored in a dry environment until use. Seedless fruit samples were ground in the grinder. Four grams of the samples was transferred to sterile Falcon tubes (15 mL), and 10 mL of ethanol was added to the tube as a solvent. This mixture was kept in an ultrasonic water bath (Kudos) for 30 min at a frequency of 35 kHz at 60 °C. Then, this reaction mixture was centrifuged for 10 min at 10,000 rpm, and the supernatants were carefully transferred to sterile Falcon tubes. The volume was made up to 10 mL with ethanol (95%). Before analysis, a portion of the supernatant was filtrated through a 0.45 µm membrane.

### 3.3. Determination of Total Phenolic Content (TP)

The total phenolic content in the ethanolic extract of five different *Berberis* samples was determined according to the Folin–Ciocalteu method suggested by Slinkard and Singleton [38]. Shortly, 680 µL of distilled water and 400 µL of 0.5 N Folin–Ciocalteau reagent were added to the samples in a tube. Then, a standard solution and 20 µL of extract were added into this mixture, and after 3 min, 400 µL of solution 10% Na_2_CO_3_ was added to the tube and the mixture was allowed to stand for 2 h with intermittent shaking. The standard concentration level was 0.03125 mg/mL^−1^ mg/mL. Finally, the absorbance was measured at 760 nm. The results were expressed as mg of gallic acid equivalent per gram of dry weight (mg GAE/g dw).

### 3.4. Determination of Total Flavonoid Content (TF)

The aluminum chloride colorimetric method for the determination of total flavonoids was modified from the procedure suggested by Fukumoto and Mazza [39]. In a test tube, 0.5 mL of fruit extracts, 0.1 mL of 10% Al(NO_3_)_3_, and 0.1 mL of 1M NH_4_.CH_3_COO were combined and incubated at room temperature for 40 min. The standard concentration level was 0.03125 mg/mL^−1^ mg/mL. The absorbance of this reaction mixture was measured at 415 nm with a UV–Vis spectrophotometer. The total flavonoid content was expressed as mg of quercetin equivalents (QE) per gram of sample (mg QE/g).

### 3.5. Condensed Tannin Content (CT) 

Condensed tannins were determined according to Julkunen-Titto’s [40] method with small modifications. Twenty-five microliters of each *Berberis* sample solution was mixed with 750 μL of 4% vanillin (prepared with MeOH), then, 375 μL of concentrated HCl was added. The well-mixed solution was incubated at room temperature in the dark for 20 min. The standard concentration level was 0.03125 mg/mL^−1^ mg/mL. The absorbance was then measured at 500 nm against a blank. The results were expressed as mg of catechin equivalents (CE) per gram of dry weight (mg CE/g DW). 

### 3.6. Antioxidant Activity

#### 3.6.1. Ferric Reducing Antioxidant Power (FRAP) Assay

The FRAP assay was carried out according to a modification by Benzie and Strain [41]. The FRAP reagent was prepared by mixing 250 mL of acetate buffer (300 mM, pH 3.6), 25 mL of TPTZ solution (10 mM TPTZ in 40 mM HCl), and 25 mL of FeCl_3_ (20 mM in water solution). For each *Berberis* spp. fruit sample and each standard, 50µL was added to 1.5 mL of freshly mixed FRAP reagent. The sample was vortexed, and all samples were incubated for 4 min. The standard concentration level was 31.25–1000 µM. The absorbance was measured at 593 nm against a control. The results were expressed as µmol FeSO_4_.7H_2_O/g.

#### 3.6.2. 1,1-Diphenyl-2-Picrylhydrazyl Radical (DPPH) Assay

The DPPH assay was performed by using the method of Molyneux [42], with some modifications. The stock solution was prepared by dissolving 4 mg DPPH with 100 mL of 100% methanol. Shortly, for each *Berberis* spp. fruit sample, a 750 µL standard solution was added to a 750 µL DPPH methanolic solution and the mixtures were shaken vigorously and left to stand in the dark for 50 min at room temperature. Then, the absorbance was read at 517 nm. Six different concentrations were used to calculate the inhibition values of the *Berberis* spp. fruit samples. To calculate DPPH radical activity, Trolox was used as a standard. 

### 3.7. Antibacterial Activity

The obtained ethanol extracts (as described in the title “extraction of plant material”) were kept at 55 °C for 48 h and the solvents were evaporated. After the removal of the solvents, the obtained active substances were weighed (128 mg) on a precision balance and transferred to 2 mL sterile Eppendorf tubes. At the end of this period, by adding DMSO to the Eppendorf tubes, the total volume was brought to 2 mL. Extracts in these Eppendorf tubes were kept at room temperature for 24 h. After vortexing, the prepared DMSO extracts were used for in vitro antibacterial activity tests.

### 3.8. Microorganisms and Growth Condition

In vitro antibacterial activities of the *Berberis*, fruit samples were determined using 10 g-positive and 8 g-negative bacteria. Selected target pathogen strains were cultured for 24 h at 37 °C using Mueller Hinton Broth (MHB, Oxoid). The suspensions were adjusted to a standard turbidity of 0.5 McFarland (106 CFU/mL) and used as inoculum [43]. The microorganisms used in the study were obtained from the Department of Medical Services and Techniques, Vocational School of Health Services, Bayburt University, Turkey.

### 3.9. Screening for Antibacterial Activity

To determine the in vitro antibacterial activity of the *Berberis* spp. fruit samples, the agar-well diffusion (AWD) method was used. For this purpose, 8 mm diameter wells were cut into the sterile Mueller Hinton Agar (MHA) mediums using a sterile cork borer [39]. After these processes, inoculums (0.5 McFarland Turbidity Standard—10^6^ CFU/mL) were seeded using sterile swabs. Next, 100 µL of DMSO extracts of the *Berberis* fruit extracts was transferred to wells and incubated at 37 °C for 24 h. Following the incubation period, the observed inhibition zones around the wells were measured with a Vernier caliper and recorded. In addition, DMSO was used as a negative control in this process. Each assay was carried out in duplicate [44].

### 3.10. Determination of Minimum Inhibition Concentration (MIC)

MIC values of *Berberis* spp. fruit extract were determined by the broth dilution method using 96-well round-bottom polystyrene microplates. For this purpose, first, 95 µL of sterile MHB was distributed to each well of the 96 well microtiter plates. Then, overnight-grown pathogenic microorganisms were adjusted to 0.5 McFarland turbidity, and 5 µL of inoculums were added to each well. As a result of these applications, 100 µL of inoculum + MHB medium solution was prepared in each well of a 96-well microplate. Then, 100 μL of extract (64 mg/mL of DMSO extracts) was added to all of the first wells and mixed at least three times. Afterward, 100 μL of the mixture was taken from the first well via a micropipette and transferred to the second well. This procedure was repeated successively up to the eighth well. In this manner, the starting concentration of *Berberis* fruit samples extracts was diluted at each step. After these applications, the absorbance values of the microplate were measured and recorded at a 600 nm wavelength (Thermo, Multiskan Go). The microplates were then incubated at 37 °C for 24 h. At the end of the incubation period, absorbance values were again measured and recorded. The first well, where the absorbance values increased, was considered as non-bactericidal or non-bacteriostatic concentrations, and the concentration of the upper wells was accepted as the MIC values [45,46].

### 3.11. Determination of Minimum Bactericidal Concentration (MBC)

After determining the MIC values of the *Berberis* spp. fruit extracts, to determine the MBC values, 10 µL suspension was removed from each well of the test microplates and transferred to the nutrient agar (NA) medium. After this process, inoculated Petri dishes were incubated at 37 °C for 24 h. At the end of this incubation period, a minimum concentration that bacterial growth was not observed was accepted as MBC. All these assays were performed twice [47].

### 3.12. Oil Extraction and Preparation of Methyl Esters

The *Berberis* spp. fruits were chosen randomly from the collected samples and separated seeds to detect the fatty acid composition. Ten grams of the dried and completely ground *Berberis* spp. seed samples was placed in the cartridge in the extractor section of the soxhlet apparatus and extracted with hexane for 6 h. The solvent of the resulting hexane–oil mixture was removed with the evaporator. To prevent hexane from remaining in the mixture obtained, it was kept in the oven for 1 h at 90 °C. When the process was completed, calculations were made by weighing the balloon, previously weighted, again and the oil content was defined. To determine the fatty acid composition of the obtained oils, 20 mg was taken and dissolved in 5 mL of hexane. To make methyl esters, 5 mL of methanol was prepared with 2N KOH and shook strongly. Gas chromatography was analyzed with a flame ionization detector (GC-FID) by taking the upper phase [48,49]. 

### 3.13. Statistical Analysis

Statistical analysis of this study was performed using the SPSS 25.0 software program. Descriptive statistics of TP, TF, CT, FRAP, DPPH, and oil content of *Berberis* spp. were determined, and Pearson correlation analysis was performed, in addition to calculating the standard deviations errors of the data. In this study, principal component analysis (PCA) was applied twice to determine both the main components of the fatty acid composition and fatty acids together with antioxidant parameters. In addition, hierarchical cluster analysis (HCA) was applied to determine the relationship between *Berberis* spp. In this study, principal component analysis (PCA) was applied two times to determine both the main components of the fatty acid composition and fatty acids together with antioxidant parameters. In addition, hierarchical cluster analysis (HCA) was applied to determine the relationship of *Berberis* spp.

## 4. Conclusions

Wild plants and medicinal aromatic plants have been used for many purposes from the past to the present. It is important to determine the fatty acid composition, antioxidant, and antimicrobial activities of these plants when consumed as food. In this study, wild *B. vulgaris* L., *B. integerrima* Bunge, *B.crataegina* DC., *B. integerrima*, the culture form of *B. vulgaris* L., and the hybrid of *B. integerrima* × *B. crataegina* were collected from Bayburt Province, Turkey. Their oil content, fatty acid compositions, antimicrobial, and antioxidant properties were detected. The oil contents, fatty acids, and phytochemical characteristics of the *Berberis* spp. fruits were affected by the variety of local breeds, species, and the collection location, considering the role of geographical conditions. The PCA and HCA results showed that the wild and cultured plants are different, and the *B. integerrima* × *B. crataegina* hybrid was diagnosed significantly different. PCA1 and PCA2 showed that fatty acids and antioxidants are principal components for *Berberis* spp. In addition, differences in fatty acids and antioxidants between hybrid and culture *Berberis* spp. support the importance of varieties. All the results obtained in this study suggest that wild *Berberis* spp. fruits collected from nature are suitable for use in the food industry.

## Figures and Tables

**Table 1:** Means and standard error (SE) of oil content and fatty acid compositions of *Berberis* spp.

		*B. crataegina* DC. (%)	*B. integerrima* × *B. crataegina* ** (%)	*B. integerrima* Bunge (%)	*B. vulgaris* * (%)	*B. vulgaris* (%)
Total Oil Content (%)		6.12 ± 1.66	7.72 ± 0.06	7.90 ± 0.11	8.57 ± 0.78	8.60 ± 0.81
Fatty acids	R.T.					
Butyric acid (C4:0)	2.89	0.12 ± 0.05	0.02 ± 0.05	0.10 ± 0.03	0.05 ± 0.01	0.04 ± 0.02
Caproic acid (C6:0)	4.73	0.21 ± 0.02	0.20 ± 0.01	0.19 ± 0.01	0.15 ± 0.04	0.21 ± 0.02
Undecanoic acid (C11:0)	14.73	0.20 ± 0.04	0.19 ± 0.02	0.14 ± 0.03	0.11 ± 0.05	0.18 ± 0.02
Lauric acid (C12:0)	17.12	0.09 ± 0.04	nd ***	0.07 ± 0.02	0.03 ± 0.01	0.05 ± 0.01
Myristic acid (C14:0)	22.67	0.22 ± 0.03	0.17 ± 0.01	0.18 ± 0.01	0.14 ± 0.04	0.21 ± 0.03
Palmitic acid (C16:0)	28.91	6.35 ± 0.73	5.75 ± 0.12	5.13 ± 0.49	5.52 ± 0.11	5.38 ± 0.25
Heptadecanoic acid (C17:0)	32.07	0.06 ± 0.01	0.68 ± 0.01	0.07 ± 0.01	0.07 ± 0.01	0.06 ± 0.01
Stearic acid (C18:0)	35.28	7.51 ± 0.69	9.20 ± 0.99	7.73 ± 0.47	7.92 ± 0.29	8.65 ± 0.45
Oleic acid (C18:1n9c)	36.61	12.85 ± 2.88	19.56 ± 3.88	14.04 ± 1.70	16.73 ± 0.99	15.49 ± 0.24
Linoleic acid (C18:2n6c)	38.97	33.26 ± 0.81	30.98 ± 1.46	34.28 ± 1.84	32.12 ± 0.33	31.60 ± 0.84
Arachidic acid (C20:0)	41.49	0.32 ± 0.11	0.44 ± 0.01	0.43 ± 0.01	0.46 ± 0.03	0.50 ± 0.07
α-Linolenic acid (C18:3n3)	41.77	37.88 ± 1.71	32.85 ± 3.31	37.09 ± 0.92	36.02 ± 0.14	36.98 ± 0.82
cis-11-Eicosenoic acid (C20:1n9)	42.72	0.17 ± 0.04	0.08 ± 0.05	0.15 ± 0.02	0.11 ± 0.02	0.14 ± 0.01
cis-11,14-Eicosadienoic acid (C20:2)	45.02	nd ***	nd ***	nd ***	nd ***	nd ***
Behenic acid (C22:0)	47.69	0.21 ± 0.03	0.27 ± 0.03	0.21 ± 0.03	0.26 ± 0.02	0.25 ± 0.01
Arachidonic acid (C20:4n6)	49.04	nd ***	nd ***	nd ***	nd ***	nd ***
Tricosanoic acid (C23:0)	50.64	nd ***	nd ***	nd ***	nd ***	nd ***
cis-5,8,11,14,17-Eicosapentaenoic acid (C20:5n3)	51.19	nd ***	0.11 ± 0.07	0.06 ± 0.02	0.06 ± 0.02	0.09 ± 0.05
Lignoceric acid (C24:0)	53.06	0.14 ± 0.10	0.13 ± 0.08	0.12 ± 0.07	0.24 ± 0.19	0.15 ± 0.11
Nervonic acid (C24:1n9)	54.09	nd ***	nd ***	nd ***	nd ***	nd ***

* Culture plant ** Hybrid *** nd: not detected.

**Table 2:** Saturated/unsaturated fatty acids ratio, saturated, unsaturated, monounsaturated, and polyunsaturated fatty acids of *Berberis* spp.

Local Breeds	Saturated Fatty Acids (%)	Unsaturated Fatty Acids (%)	Saturated/Unsaturated Fatty Acid Ratio (%)	Monounsaturated Fatty Acids (%)	Polyunsaturated Fatty Acids (%)
*B. crataegina* DC.	15.84	84.16	1.88	13.03	71.13
*B. integerrima* × *B. crataegina* **	16.42	83.58	1.96	19.64	63.94
*B. integerrima* Bunge	14.38	85.62	1.68	14.19	71.43
*B. vulgaris* L. *	14.97	85.03	1.76	16.84	68.19
*B. vulgaris* L.	15.70	84.30	1.86	15.63	68.66

* Culture plant ** Hybrid.

**Table 3:** Minimum inhibition concentration (mg/mL), minimum bactericidal concentration (mg/mL) and inhibition zone diameter (mm) of *Berberis* spp.

	Microorganisms	*B. crataegina* DC.			*B. integerrima* × *B. crataegina*			*B. integerrima* Bunge			*B. vulgaris* L. (Culture)			*B. vulgaris* L. (Wild)		
		IZD	MIC	MBC	IZD	MIC	MBC	IZD	MIC	MBC	IZD	MIC	MBC	IZD	MIC	MBC
**Gram positive**	B1	13	-	-	27	16	32	29	16	32	25	32	32	27	32	32
	B2	14	-	-	30	16	32	32	8	32	29	16	32	35	8	16
	B3	17	-	-	29	16	32	33	8	32	30	16	16	39	4	8
	B4	14	-	-	28	16	32	30	16	32	34	8	16	36	8	16
	B5	-	-	-	25	32	-	19	-	-	26	32	32	30	16	32
	B6	-	-	-	20	32	-	21	32	-	23	32	-	24	32	-
	B7	-	-	-	19	32	-	22	32	-	21	32	-	27	16	32
	B8	-	-	-	21	32	-	29	16	32	24	32	-	32	16	32
	B9	13	-	-	26	32	32	23	32	32	26	32	32	26	32	32
	B10	12	-	-	23	32	-	27	16	32	24	32	32	28	16	32
**Gram negative**	B11	13	-	-	34	8	32	33	8	32	35	8	16	37	4	16
	B12	12	-	-	21	32	-	25	32	32	24	32	32	28	16	32
	B13	11	-	-	18	-	-	20	-	-	19	-	-	23	32	-
	B14		-	-	20	-	-	17	-	-	18	-	-	21	32	-
	B15		-	-	18	-	-	18	-	-	20	32	-	20	32	-
	B16	-	-	-	20	32	-	19	32	-	19	-	-	22	32	-
	B17	-	-	-	34	8	32	37	4	16	32	16	32	39	4	8
	B18	-	-	-	21	32	-	25	32	32	21	32	-	31	16	32

Minimum inhibition concentration (MIC) and minimum bactericidal concentration (MBC) results. Inhibition zone diameter (IZD). BEE: *Berberis* ethanolic extract; Microorganisms: B1: *Bacillus cereus* ATCC 14579; B2: *Bacillus cereus* BC 6830; B3: *Enterococcus faecalis* ATCC 49452; B4: *Enterococcus faecalis* NCTC 12697; B5: *Enterococcus faecium* ATCC 700211; B6: *Staphylococus aureus* ATCC 25923; B7: *Staphylococcus aureus* NCTC 10788; B8: *Staphylococcus aureus* BC 7231; B9: *Streptococcus mutans* ATCC 35668; B10: *Streptococcus salivarus* ATCC 13419; B11: *Acinetobacter baumannii* ATCC BA 1609; B12: *Escherichia coli* ATCC BAA 25-23; B13: *Escherichia coli* NCTC 9001; B14: *Escherichia coli* BC 1402; B15: *Pseudomonas aeruginosa* ATCC 9070; B16: *Pseudomonas aeruginosa* NCTC 12924; B17: *Salmonella Typhimurium* RSSK 95091; B19: *Yersinia enterocolitica* ATCC 27729.

**Table 4:** Means and standard error (SE), total phenolic content, total flavonoid content, condensed tannin content, and antioxidant activity *Berberis* spp. fruits.

Local Breeds	TP mg GAE/g	TF mg QE/g	CT mg CE/g	FRAP (μmol FeSO_4_.7H_2_O/g)	DPPH SC_50_ mg/mL
*B. crataegina* DC.	10.84 ± 4.35	2.20 ± 0.49	6.92 ± 0.01	236.35 ± 4.64	0.36 ± 0.01
*B. integerrima* × *B. crataegina* **	16.10 ± 1.71	0.46 ± 0.03	2.93 ± 0.01	218.55 ± 2.46	0.20 ± 0.01
*B. integerrima* Bunge	17.28 ± 2.23	0.41 ± 0.01	2.00 ± 0.03	305.29 ± 25.08	0.20 ± 0.00
*B. vulgaris* L. *	15.37 ± 1.52	0.45 ± 0.01	1.75 ± 0.01	349.52 ± 1.49	0.14 ± 0.01
*B. vulgaris* L.	28.92 ± 1.94	0.56 ± 0.14	1.97 ± 0.01	621.02 ± 25.03	0.10 ± 0.01
**Trolox**					0.004 ± 0.00

* Culture plant ** Hybrid.

**Table 5:** Eigenvalues and percentage of variance for investigated parameters of PCA analysis.

		PC1	PC2	PC3	PC4
**PCA-1**	Eigenvalue	11.59	6.95	2.30	1.16
	Variability (%)	52.69	31.60	10.43	5.28
	Cumulative (%)	52.69	84.28	94.72	100.00
**PCA-2**	Eigenvalue	10.86	4.87	3.68	1.58
	Variability (%)	51.73	23.21	17.54	7.52
	Cumulative (%)	51.73	74.94	92.48	100.00

## Data Availability

Not available.

## Sample Availability

Samples of the compounds are not available from the authors.
